# From the Cochrane Library: Interventions for the Prevention of Herpes Simplex Labialis (Cold Sores on the Lips)

**DOI:** 10.2196/38322

**Published:** 2022-06-14

**Authors:** Torunn Sivesind, Jennifer Viola, Linda Zhang, Robert Dellavalle, Ching-Chi Chi

**Affiliations:** 1 Department of Dermatology University of Colorado Anschutz Medical Campus Aurora, CO United States; 2 Heritage College of Osteopathic Medicine Ohio University Athens, OH United States; 3 Ochsner Clinical School School of Medicine University of Queensland New Orleans, LA United States; 4 Dermatology Service Rocky Mountain Regional Veterans Affairs Medical Center Aurora, CO United States; 5 Department of Dermatology Linkou Chang Gung Memorial Hospital Taoyuan Taiwan

**Keywords:** cold sore, fever blister, herpes labialis, secondary prevention, herpes simplex, dermatology, herpes, antiviral resistance

In 2016, the World Health Organization estimated that 67% of the global population is infected with herpes simplex virus type 1 (HSV-1), which causes herpes simplex labialis (HSL) [1].The lifetime prevalence of recurrent HSL is 20% to 52.5% [2].It is highly contagious and mainly transmitted through oral-to-oral contact [1]. HSL is a lifelong, often asymptomatic infection that lays dormant in the trigeminal nerve. Common symptoms include prodromal tingling or burning sensation around the mouth and eruption of painful, self-limiting vesicles (“cold sores”) progressing to unsightly crusts [1,2]. HSV-1 recurrence can be triggered by ultraviolet light, stress, premenstrual changes, and surgical procedures; its highly visible nature can lead to embarrassment and psychological distress [2]. Antiviral medications are the standard treatment but have adverse effects such as rash, headache, and gastrointestinal upset [1].

A 2015 Cochrane review [2] assessed the effects of preventative interventions for HSL in immunocompetent people of all ages, analyzing evidence from 32 randomized controlled trials on 19 preventative measures. Primary outcomes and key findings are summarized in Table 1.

**Table 1:** Treatment comparison from the Cochrane review [2] for herpes simplex labialis (HSL) with respective results, risk ratio (RR) with CI, comparative risk (CR) with or without *P* value, or mean difference (MD) with CI.

Comparison	Measurement (primary outcome)	Result	Statistical results	Quality of evidence
Oral acyclovir vs placebo (short term ≤1 month): (1) 800 mg 2×/day; (2) 400 mg 2×/day; (3) 200 mg 5×/day	Incidence of HSL during use of the preventive intervention	Unclear. No preventative effect; not currently recommended	(1) RR 1.08 (0.62 to 1.87) (2) RR 0.26 (0.13 to 0.51) (3) RR 0.46 (0.20 to 1.07)	(1) Moderate (2) Low (3) Low
Oral acyclovir vs placebo (long term >1 month): 400 mg 2×/day	Incidence of HSL during use of the preventive intervention (clinical recurrences)	Acyclovir was slightly superior. Recommended (small effect)	CR 0.85 vs 1.80 episodes per participant per 4-month period MD –3.6 (–7.2 to 0)	Low
Oral valaciclovir vs placebo (short term ≤1 month): 2 g 2×/day for the first day, 1 g 2×/day for the second day	Incidence of HSL during use of the preventive intervention	No significant difference. No preventative effect; not currently recommended	RR 0.55 (0.23 to 1.28)	Moderate
Oral valacyclovir vs placebo (long term >1 month): 500 mg 1×/day	Incidence of HSL during use of the preventive intervention	Valacyclovir was slightly superior. Recommended (small effect)	CR 0.12 vs 0.21 episodes per participant per month MD 0.009	Moderate
Oral famciclovir vs placebo (short term ≤1 month): (1) 125 mg 3×/day; (2) 250 mg 3×/day; (3) 500 mg 3×/day	Incidence of HSL during use of the preventive intervention	No significant difference. No preventative effect; not currently recommended	(1) RR 0.74 (0.5 to 1.11) (2) RR 0.69 (0.45 to 1.04) (3) RR 0.82 (0.56 to 1.21)	(1) Moderate (2) Moderate (3) Moderate
Oral levamisole vs placebo (long term >1 month): 2.5 mg/kg 2×/week	Incidence of HSL during use of the preventive intervention	No consistent data. No preventative effect; not currently recommended	MD –2 (–2.24 to –1.76)	Very low
Oral lysine vs placebo (long term >1 month): 1000 mg 1×/day	Incidence of HSL during use of the preventive intervention	No significant difference. No preventative effect; not currently recommended	MD –0.04 (–0.37 to 0.29)	Very low
Topical acyclovir 5% cream vs placebo (short term ≤1 month): 5×/day	Incidence of HSL during use of the preventive intervention	No significant difference. No preventative effect; not currently recommended	RR 0.91 (0.48 to 1.72)	Moderate
Topical acyclovir 5% and 348U87 3% cream vs placebo (short term ≤1 month): 1×/2 hours during awake hours	Incidence of HSL during use of the preventive intervention (by culture)	No significant difference. No preventative effect; not currently recommended	RR 0.78 (0.19 to 3.14)	Very low
Topical foscarnet 3% vs placebo (short term ≤1 month): 8×/day	Incidence of HSL during use of the preventive intervention	No significant difference. No preventative effect; not currently recommended	RR 1.08 (0.82 to 1.4)	Moderate
Topical 1,5 pentanediol vs placebo (long term >1 month): 2×/day	Incidence of HSL during use of the preventive intervention	No significant difference. No preventative effect; not currently recommended	CR 120 episodes out of 53 (topical) vs 109 episodes out of 50 (placebo);*P*>.05	Moderate
Sunscreen vs placebo (short term ≤1 month); 1× prior to immediate exposure to (1) solar radiation and (2) experimental ultraviolet light	Incidence of HSL during use of the preventive intervention	Unclear. Not currently recommended; further research warranted	(1) Under sunlight: RR 1.13 (0.25 to 5.06) (2) Under experimental ultraviolet light: RR 0.07 (0.01 to 0.33)	(1) Low (2) Very low
Interferon injection (70,000 U/kg) vs placebo (short term ≤1 month): (1) presurgical 2×/day; (2) postsurgical 2×/day; (3) pre- and postsurgical 2×/day	Incidence of HSL during use of the preventive intervention	Unclear. No preventative effect; not currently recommended	(1) RR 1.59 (1.05 to 2.41) (2) RR 0.99 (0.59 to 1.66) (3) RR 0.57 (0.34 to 0.95)	(1) Low (2) Low (3) Low
Gamma globin injection vs histamine (control, dilute 1:5000) (short term ≤1 month): 0.2 ml 1× dose	Duration of HSL outbreak	No significant difference. No preventative effect; not currently recommended	MD 0.7 (–0.55, 1.95)	Low
Thymopentin injection vs placebo (long term >1 month): 50 mg 3×/week	Incidence of HSL during use of the preventive intervention	Thymopentin was superior. Not currently recommended; further research warranted	CR median 0.2 for thymopentin vs 0.9 for placebo;*P*=.0027	Moderate
Herpes simplex virus type I vaccine injection vs placebo (short term ≤1 month): 1× dose	Incidence of HSL during use of the preventive intervention	No significant difference. No preventative effect; not currently recommended	CR 1.6 vs 1.3 recurrences in 4 months (*P*=.1)	Moderate
Laser (low intensity, 690 nm, 80 mW/cm^2^, 48 J/cm^2^) vs no intervention (short term ≤1 month): 1×/day	Time to first occurrence	Low-intensity diode laser was superior but low-energy gallium-aluminum-arsenide laser was not. Not currently recommended; further research warranted	Low-energy gallium-aluminum-arsenide laser: CR 0.076 vs 0.116 recurrences per month (*P*=.076) Low-intensity diode laser, median recurrence-free interval: MD 30 (21.42 to 38.58)	Very low
Hypnotherapy vs control (long term >1 month): 1×/week	Change in the frequency of recurrence	Hypnotherapy was superior. Not currently recommended; further research warranted	MD –6.5 (–8.76 to –4.24)	Very low

Compared to the placebo, long-term oral acyclovir and valaciclovir reduced recurrences, although clinical benefit is limited. Limited data suggest thymopentin, low-level laser therapy (LLLT), and hypnotherapy may be effective, but further research is required. There was no evidence supporting the efficacy of lysine, LongoVital supplementation, gamma globulin, the HSV vaccine, the yellow fever vaccine, levamisole, or interferon. Compared to the placebo, there was no significant increase in adverse effects for any of the interventions assessed.

Further research is needed to establish the safety and efficacy of other preventive methods, such as HSV-1 subunit and dendritic cell–based vaccines, LLLT, and topical corticosteroids [1]. A dendritic cell vaccine pilot study (n=14) reported a 3-fold reduction in recurrence during the posttreatment period [3]. Laser therapy relies on analgesic, anti-inflammatory, anti-infective, and biostimulating effects, promoting tissue regeneration and immune response. Although LLLT is promising, caution is warranted due to heterogenicity in study methods and laser parameters [4].

This Cochrane review [2] confirms the preventative efficacy of long-term oral antivirals, highlights the need for further research on sunscreen and natural sunlight, and emphasizes the importance of defining core outcome sets for future studies to adopt. Establishing additional preventative options for HSL remains of paramount importance, considering its significant disease burden and growing antiviral resistance.

## Abbreviations

HSL: herpes simplex labialis
HSV-1: herpes simplex virus type 1
LLLT: low-level laser therapy
